# *Ex vivo* vs. *in vivo* antibacterial activity of two antiseptics on oral biofilm

**DOI:** 10.3389/fmicb.2015.00655

**Published:** 2015-07-02

**Authors:** Isabel Prada-López, Víctor Quintas, Maria A. Casares-De-Cal, Juan A. Suárez-Quintanilla, David Suárez-Quintanilla, Inmaculada Tomás

**Affiliations:** Oral Sciences Research Group, Special Needs Unit, School of Medicine and Dentistry, University of Santiago de CompostelaSantiago de Compostela, Spain

**Keywords:** antiseptic, chlorhexidine, essential oils, immersion, mouthwash, PL-biofilm

## Abstract

**Aim:** To compare the immediate antibacterial effect of two application methods (passive immersion and active mouthwash) of two antiseptic solutions on the *in situ* oral biofilm.

**Material and Methods:** A randomized observer-masked crossover study was conducted. Fifteen healthy volunteers wore a specific intraoral device for 48 h to form a biofilm in three glass disks. One of these disks was used as a baseline; another one was immersed in a solution of 0.2% Chlorhexidine (0.2% CHX), remaining the third in the device, placed in the oral cavity, during the 0.2% CHX mouthwash application. After a 2-weeks washout period, the protocol was repeated using a solution of Essential Oils (EO). Samples were analyzed for bacterial viability with the confocal laser scanning microscope after previous staining with LIVE/DEAD® BacLight™.

**Results:** The EO showed a better antibacterial effect compared to the 0.2% CHX after the mouthwash application (% of bacterial viability = 1.16 ± 1.00% vs. 5.08 ± 5.79%, respectively), and was more effective in all layers (*p* < 0.05). In the immersion, both antiseptics were significantly less effective (% of bacterial viability = 26.93 ± 13.11%, EO vs. 15.17 ± 6.14%, 0.2% CHX); in the case of EO immersion, there were no significant changes in the bacterial viability of the deepest layer in comparison with the baseline.

**Conclusions:** The method of application conditioned the antibacterial activity of the 0.2% CHX and EO solutions on the *in situ* oral biofilm. The *in vivo* active mouthwash was more effective than the *ex vivo* passive immersion in both antiseptic solutions. There was more penetration of the antiseptic inside the biofilm with an active mouthwash, especially with the EO. Trial registered in clinicaltrials.gov with the number NCT02267239. URL: https://clinicaltrials.gov/ct2/show/NCT02267239.

## Introduction

The use of oral antiseptics is a recommended procedure for the chemical control of the oral biofilms. These oral antiseptics may kill the microorganisms, reduce bacterial virulence, and retard the dental plaque formation. Due to this action on the bacteria forming dental biofilms, a reduction of the oral disease is expected after their application (Corbin et al., 2011).

Among dental practitioners, the most commonly prescribed oral antiseptics have been those including in their formulation Chlorhexidine (CHX) (Varoni et al., 2012) or Essential Oils (EO) (Axelsson, 2004). Although the antimicrobial effectiveness of both has been shown in previous studies (Gunsolley, 2010; Quintas et al., 2015), they exhibit different traits when certain methodologies are followed, which, in some cases, may limit the reliability of the results. Some of these differences have been recognized by the scientific community, who cautiously interpret the results of the studies that have used an *in vitro*-formed biofilm (Auschill et al., 2004). The use of determinate bacteria to create a biofilm in an artificial environment may result in a measure of antiseptic effectiveness that may not be representative of the *in situ* situation (Auschill et al., 2005).

Bacteria living in dental plaque develop relationships forming an extracellular matrix which is a highly resistant three-dimensional (3-D) structure. This association makes the biofilm bacteria from 10 to 1000 times more resistant to an antiseptic (Fine et al., 2001; Davies, 2003). Given this, the handling of the *in situ* formed biofilm is an important characteristic that studies involving oral biofilms should take into account. In some studies the 3-D structure of the oral biofilm is altered when the sample is collected (Pan et al., 2000; Fine et al., 2005) or during the analysis process (Jentsch et al., 2002; Vitkov et al., 2005). The distortion of the 3-D structure probably influences the quantification of the antiseptic effectiveness. To avoid this, specific oral devices have been designed to allow the formation of a non-disturbed biofilm, similar to the dental plaque, which has been called plaque-like biofilm (PL-biofilm) (Prada-López et al., 2015a; Quintas et al., 2015). Furthermore, the use of confocal laser scanning microscope (CLSM) has allowed the possibility to analyse the *in situ* biofilm in their natural hydrated state without losing its complex structure (Arweiler et al., 2004). The CLSM has been used in combination with dual live/dead staining solutions. This has given to investigators the possibility to analyse the viability of a non-disturbed *in situ* oral biofilm, before and after the application of the antiseptics. Among the different available staining solutions, the SYTO 9 and propidium iodide has been one of the most successfully employed combinations of fluorochromes for visualizing dental plaque (Fuchslocher Hellemann et al., 2013; Hannig et al., 2013; Tawakoli et al., 2013; Prada-López et al., 2015a; Quintas et al., 2015).

Recently, some authors have stated that the methodology of the application of a given antiseptic could be an important factor which might condition the results on oral antiseptic effectiveness (Quintas et al., 2015). Commonly, in studies with undisturbed biofilm, the application of the antiseptic has been an *ex vivo* immersion of the sample into the solution (Zaura-Arite et al., 2001; Dong et al., 2010; Gosau et al., 2010; von Ohle et al., 2010). However, in recent series about the bacterial effect of CHX and EO, the participants have undergone an active mouthwash with the antiseptic *in vivo* (García-Caballero et al., 2013; Quintas et al., 2015), differing from the previously described results, mainly in regard to the EO activity.

Based on these previous findings, the authors of the present study intended to assess if the methodology of the antiseptic application might condition the obtained results in terms of bacterial viability of the PL-biofilm. Therefore, the objective of the present study was to compare the immediate effect of two antiseptic application methods, using separate solutions of 0.2% CHX and EO applied either by a passive immersion or an active mouthwash (*ex vivo* vs. *in vivo* exposure).

## Material and methods

The present study was designed as a randomized, observer-masked, crossover study. The immediate effect of 0.2% CHX and EO solutions was tested using them separately in immersion and mouthwash application on an *in situ* model of PL-biofilm growth. The supporting CONSORT checklist is available as supporting information (supplementary Table 1). This project got the approval of the Clinical Research Ethics Committee of Galicia (number 2012/393) and registered in clinicaltrials.gov with the number NCT02267239. URL: https://clinicaltrials.gov/ct2/show/NCT02267239.

### Selection of the study group

**Figure 1:**
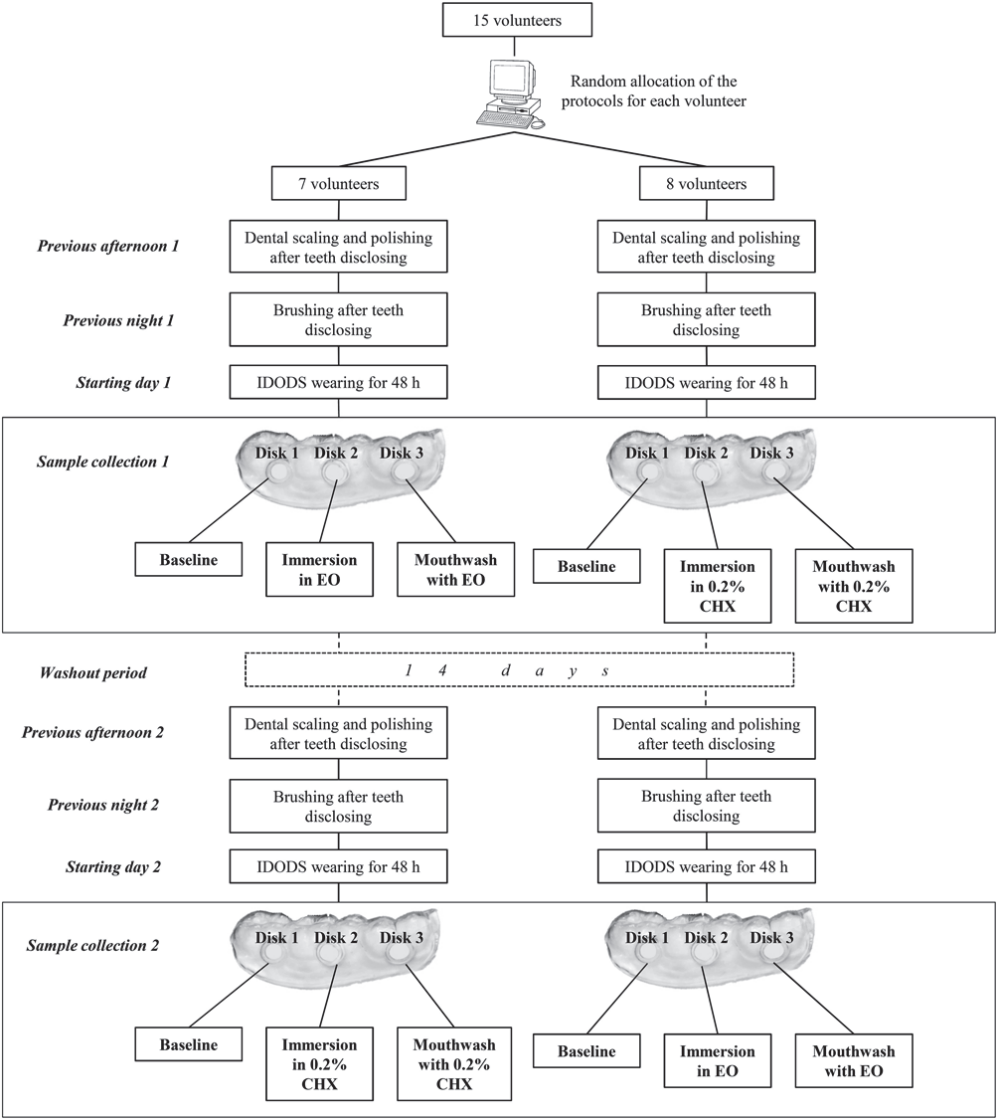
**Protocol of the study**.

To calculate an a priori sample size, the following statistical criteria were established: an effect size of 0.35, an alpha error of 0.05 and a statistical power of 87%. Assuming these criteria and using the repeated measures ANOVA test, a sample size of 15 subjects was required. The sample size calculation was performed using the program G*Power 3.1.5. The participants were recruited among dental students at the Faculty of Medicine and Dentistry of Santiago de Compostela (Spain), where volunteer enrolment was asked by responding to advertisements for the participation in a research study at the faculty hall. All of these volunteers were revised by the same trained clinician to ensure they fulfilled all inclusion and exclusion criteria. The volunteers chosen met the same inclusion and exclusion criteria of previous publications of our group (García-Caballero et al., 2013; Prada-López et al., 2015a; Quintas et al., 2015). The inclusion criteria were the following: being systemically healthy adult volunteers between 20 and 45 years old, who presented a good oral health status: a minimum of 24 permanent teeth with no evidence of gingivitis or periodontitis (Community Periodontal Index score = 0) (WHO, 1997) and an absence of untreated caries at the beginning of the study. The following exclusion criteria were applied: smoker or former smoker, presence of dental prostheses or orthodontic devices, antibiotic treatment or routine use of oral antiseptics in the previous 3 months, and presence of any systemic disease that could alter the production or composition of saliva. Before the start of each phase, a full mouth scaling with ultrasonic instruments and teeth polishing with rubber cup after dental disclosure was performed by the same trained clinician on all selected participants (Figure 1). Written informed consent was obtained from all participants in the study.

### Intraoral device of overlaid disk-holding splints (IDODS) for biofilm *in situ* formation

**Figure 2:**
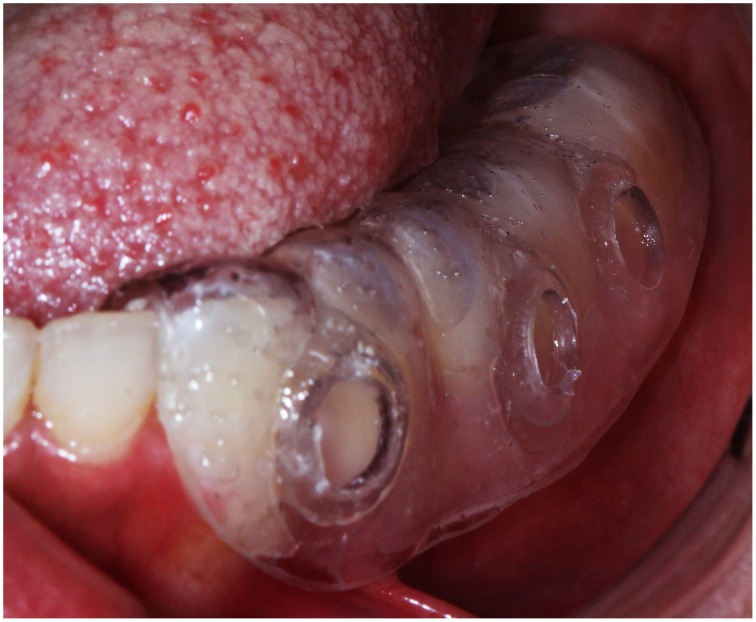
**Intraoral view of the Intraoral Device Overlaid Disk-holding Splint (IDODS)**.

Some *in situ* models for the growth of the biofilm have been previously described (Netuschil et al., 1998; Auschill et al., 2001, 2005; Arweiler et al., 2004). After their consideration, an individualized splint of a lower hemi-arch was created for each volunteer, following the same protocol given in previous studies (García-Caballero et al., 2013; Prada-López et al., 2015a,b; Quintas et al., 2015). The Intraoral Device of Overlaid Disk-holding Splints (IDODS) worn by the volunteers held three glass disks (6 mm in diameter, 1 mm thickness); these were polished at 800 grit. This splint has been already described in previous own works (Prada-López et al., 2015a,b; Quintas et al., 2015) (Figure 2).

The IDODS with the glass disks was worn by the subjects for 48 h (2 days) to favor growth of the PL-biofilm. They were allowed to withdraw it from the oral cavity only during meals and to perform oral hygiene measures (while it had to be stored in a previously provided opaque container in humid conditions). In order to not to disturb the growing of the PL-biofilm, volunteers could not use any toothpaste or mouthwash as a complement for the mechanical removal of bacterial plaque.

### Application of the 0.2% CHX and EO to PL-biofilm

The sample analysis was divided into two phases, each of following the application protocol of the antiseptic. The first consisted of withdrawing the glass disks one by one from the splint (Figure 1) after the volunteer had worn it for 48 h. The distal of these disks was used as a baseline sample. The second disk underwent one of two protocols:

A single, 30-s immersion in 1 mL of 0.2% Chlorhexidine (Oraldine Perio®, Johnson & Johnson, Madrid, Spain) (Im-0.2% CHX).

-OR-

A single, 30-s immersion in 1 mL of Essential Oils in a hydroalcoholic solution (Listerine Mentol, Listerine®, Johnson & Johnson, Madrid, Spain) (Im-EO).

Next, the second phase of the study was conducted. The last disk in the splint, placed in the oral cavity, was withdrawn after the volunteer performed the following under supervision:

A single, 30-s mouthwash with 10 mL of 0.2% Chlorhexidine (Oraldine Perio®, Johnson & Johnson, Madrid, Spain) (Mw-0.2% CHX), following the instructions of the manufacturer.

-OR-

A single, 30-s mouthwash with 20 mL of Essential Oils in a hydroalcoholic solution (Listerine Mentol, Listerine®, Johnson & Johnson, Madrid, Spain) (Mw-EO), following the instructions of the manufacturer.

Using an internet-based balanced randomization system (www.randomization.com) that indicated the antiseptic each subject would use first and second, as well as the hemi-arch (left or right) selected for the immersion and mouthwash. All subjects performed the two tests with a rest period of 14 days in-between (Figure 1).

### Collection of the samples of PL-biofilm

On the day of the experiment, the volunteers were not allowed to eat or drink during the course of the tests. PL-biofilm samples collection was done individually (samples were taken from just one volunteer per day), starting at 8.30 AM (first baseline sample and immersions) and finished at 9.30 AM (mouthwash).

Immediately after the glass disks were withdrawn from the splints, they were submerged in 100 μL of fluorescence solution LIVE/DEAD® BacLight™ and kept in a dark chamber at room temperature for 15 min. A single investigator, masked to the study design, performed the microscopic observation using a Leica TCS SP2 laser scanning spectral confocal microscope (Leica Microsystems Heidelberg GmbH, Mannheim, Germany) with an HCX APOL 63x/0.9 water-immersion lens.

### Processing of the PL-biofilm samples

In the present series, the same protocol described by Quintas et al. (2015) was followed to evaluate the different fields within the disks. Four selected fields (considered as representative of the whole sample), which were in the central part of each disk were evaluated; their mean measures of the thickness and bacterial viability represented the whole sample thickness and bacterial viability, respectively. The maximum biofilm thickness of each field was divided into three equivalent zones or same sized layers: outer layer (layer 1), middle layer (layer 2) and inner layer (layer 3).

**Figure 3:**
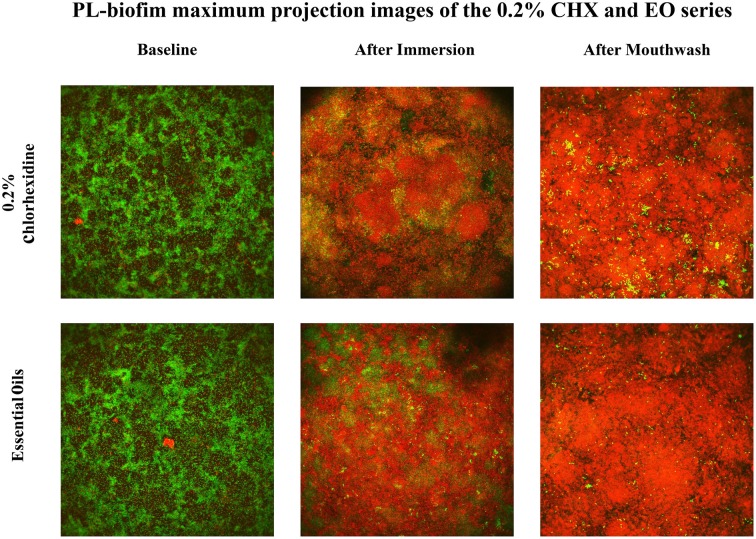
**Representative images of the PL-biofilm (“stacked projection” of images in the “Z” axis) bacterial viability under basal conditions, after immersion and after mouthwash with 0.2% Chlorhexidine and Essential Oils**. (They are images of representative fields of the PL-biofilm. It is a maximum projection of all obtained images in the plane XY in the Z axis for a same field. That is commonly called “stacked projection.” These images do not represent nor the outer, the middle or the inner layers, they represent all of them projected in the same axis).

The capture of the data was done with the same settings in all cases, according to previously presented parameters (Quintas et al., 2015) (Figure 3).

Quantification of bacterial viability was also done as previously presented (Quintas et al., 2015) using the cytofluorographic analysis (Leica Confocal Software) of XY images. In this analysis, the images of each fluorochrome were defined as “channels” (SYTO 9 occupies the green channel and propidium iodide the red channel). Square capture masks were used to measure the area occupied (μm^2^) by the pixels in each channel, determining the total area occupied by the biofilm and the corresponding percentage of viability. The intensity range was considered a positive signal if it was between 100 and 255. Determination of the mean percentage of bacterial viability in each field required sections with a minimum area of biofilm of 250 μm^2^; the mean percentage of bacterial viability of the biofilm was calculated for the corresponding sample and for each biofilm layer.

### Statistical analysis

The data on thickness and bacterial viability in the PL-biofilm, were expressed as mean and standard deviation of the mean. The type of distribution of the quantitative variables analyzed was determined using the Kolmogorov–Smirnov test, obtaining a normal distribution for all values. Repeated measures ANOVA test and pairwise comparisons (with the Bonferroni correction) were used for the analysis of intra- and inter-application results for 0.2% CHX and EO and inter-antiseptic solution results (including differentiating between the 3 biofilm layers). Measurements were statistical significant if the *p* value less than 0.05. The statistical analysis was performed by the PASW Statistics Base 20 package for Windows (IBM, Madrid, Spain).

## Results

### Influence of the application of methods of 0.2% CHX and EO on the PL-biofilm thickness

The thicknesses obtained in both baseline disks were 19.17 and 20.33 μm, before applying either 0.2% CHX or EO, respectively. After the Im-0.2% CHX, the thickness was 17.64 μm and 15.77 μm after the mouthwash. When the applied antiseptic was the EO, the obtained thicknesses were 17.97 and 20.82 μm, after immersion and mouthwash, respectively. No significant differences were found in either case.

### Influence of the application methods of 0.2% CHX and EO on the PL-biofilm bacterial viability

The bacterial viability in the baseline disks was not significantly different between the two series of 0.2% CHX and the EO (72.21 ± 10.48% vs. 75.72 ± 14.33%).

**Figure 4:**
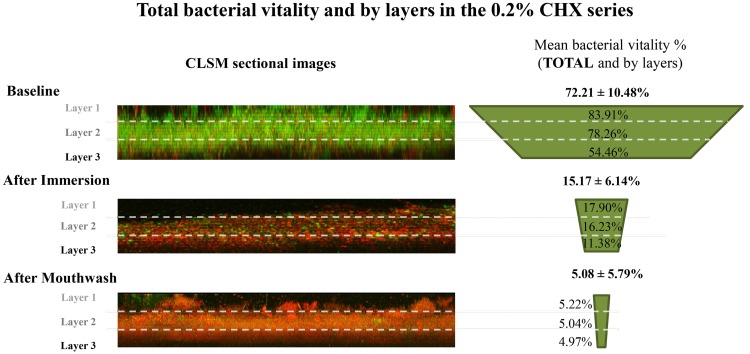
**Total bacterial viability and by PL-biofilm layers in the 0.2% Chlorhexidine series**. (PL-biofilm, plaque like-biofilm; 0.2% CHX, 0.2% of Chlorhexidine; CLSM, confocal laser scanning microscope).

After the Im-0.2% CHX, the bacterial viability was significantly reduced to 15.17 ± 6.14%. In contrast, the bacterial viability after the Mw-0.2% CHX was 5.08 ± 5.79% (Figure 4), which was significantly lower than the Im-0.2% CHX (*p* = 0.001). In addition, both results differed significantly from their baseline values (*p* < 0.001) (Table 1).

**Table 1:** **Inter-application analysis for 0.2% of Chlorhexidine and Essential Oils by layers**.

**Application method (inter-application)**	**0.2% Chlorhexidine**	**Essential Oils**
**TOTAL**		
Baseline vs. immersion	*p* < 0.001	*p* < 0.001
Baseline vs. mouthwash	*p* < 0.001	*p* < 0.001
Immersion vs. mouthwash	*p* = 0.001	*p* < 0.001
**LAYER 1**		
Baseline vs. immersion	*p* < 0.001	*p* < 0.001
Baseline vs. mouthwash	*p* < 0.001	*p* < 0.001
Immersion vs. mouthwash	*p* = 0.001	*p* = 0.002
**LAYER 2**		
Baseline vs. immersion	*p* < 0.001	*p* < 0.001
Baseline vs. mouthwash	*p* < 0.001	*p* < 0.001
Immersion vs. mouthwash	*p* = 0.001	*p* < 0.001
**LAYER 3**		
Baseline vs. immersion	*p* < 0.001	–
Baseline vs. mouthwash	*p* < 0.001	*p* < 0.001
Immersion vs. mouthwash	*p* = 0.046	*p* < 0.001

**Figure 5:**
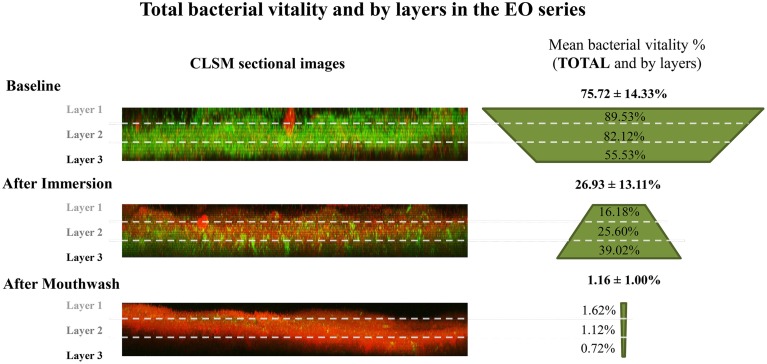
**Total bacterial viability and by PL-biofilm layers in the Essential Oils series**. (PL-biofilm, plaque like-biofilm; EO, Essential Oils; CLSM, confocal laser scanning microscope).

In the same way, the Im-EO significantly reduced the bacterial viability to 26.93 ± 13.11%. However, after Mw-EO, the bacterial viability was reduced to 1.16 ± 1.00% (Figure 5), which was significantly lower than the Im-EO (*p* < 0.001). Besides, both results differed significantly from their baseline values (*p* < 0.001) (Table 1).

In the comparison of the two antiseptics, the Im-0.2% CHX obtained significantly lower values of bacterial viability compared with the Im-EO (15.17 ± 6.14% CHX vs. 26.93 ± 13.11% EO, *p* < 0.05). On the other hand, the Mw-EO achieved significantly lower bacterial viability in comparison with the Mw-0.2% CHX (1.16 ± 1.00% vs. 5.08 ± 5.79%, *p* < 0.05) (Table 2).

**Table 2:** **Inter-antiseptic solution analysis between 0.2% of Chlorhexidine and Essential Oils by layers**.

**Application method (inter-antiseptic solution)**	**0.2% Chlorhexidine vs. Essential Oils**
**BASELINE**	
Total	–
Layer 1	–
Layer 2	–
Layer 3	–
**IMMERSION**	
Total	*p* = 0.007
Layer 1	–
Layer 2	–
Layer 3	*p* < 0.001
**MOUTHWASH**	
Total	*p* = 0.020
Layer 1	*p* = 0.041
Layer 2	*p* = 0.034
Layer 3	*p* = 0.006

### Influence of the application methods of CHX and EO on the PL-biofilm bacterial viability by layers

When accounting for the different layers, the values for baseline bacterial viability decreased progressively for deeper layers (baseline sample prior to 0.2% CHX application in layer 1 = 83.91 ± 9.51%, layer 2 = 78.26 ± 9.93%, and layer 3 = 54.46 ± 25.43% (Figure 4 and Table 3); baseline sample prior to EO application in layer 1 = 89.53 ± 8.34%, layer 2 = 82.12 ± 13.26%, and layer 3 = 55.53 ± 28.19%) (Figure 5 and Table 3).

**Table 3:** **Intra-application analysis for 0.2% of Chlorhexidine and Essential Oils by layers**.

**Application method (intra-application)**	**0.2% Chlorhexidine**	**Essential Oils**
**BASELINE**		
Layer 1 vs. Layer 2	*p* = 0.038	*p* = 0.021
Layer 1 vs. Layer 3	*p* = 0.005	*p* = 0.001
Layer 2 vs. Layer 3	*p* = 0.006	*p* = 0.001
**IMMERSION**		
Layer 1 vs. Layer 2	*–*	*p* = 0.004
Layer 1 vs. Layer 3	*–*	*p* < 0.001
Layer 2 vs. Layer 3	*–*	*p* < 0.001
**MOUTHWASH**		
Layer 1 vs. Layer 2	*–*	*p* = 0.021
Layer 1 vs. Layer 3	*–*	*–*
Layer 2 vs. Layer 3	*–*	*–*

After the Im-0.2% CHX protocol, the sample showed a homogeneous decrease in value from the baseline situation in all layers (Im-0.2% CHX, layer 1 = 17.90 ± 9.16%, layer 2 = 16.23 ± 7.14%, and layer 3 = 11.38 ± 5.81%, *p* < 0.001 in all cases). In addition, after the Mw-0.2% CHX protocol, the bacterial viability was more reduced compared to the immersion protocol (Mw-0.2% CHX, layer 1 = 5.22 ± 6.16%, layer 2 = 5.04 ± 6.40%, layer 3 = 4.97 ± 5.00%, *p* < 0.05 comparing Im-0.2% CHX and its baseline) (Figure 4 and Table 1).

After the Im-EO protocol, the sample showed a general decrease from the baseline disk in the superficial layers (Im-EO, layer 1 = 16.18 ± 12.38% and layer 2 = 25.60 ± 14.51%, *p* < 0.001 in layers 1 and 2) but not in the deepest layer (Im-EO, layer 3 = 39.02 ± 17.50%). In contrast, the results after the Mw-EO showed a highly reduced bacterial viability in all layers of the PL-biofilm, thus it was significantly more effective at reducing bacterial viability than the immersion protocol (Mw-EO, layer 1 = 1.62± 1.54%, layer 2 = 1.12 ± 1.16%, and layer 3 = 0.72 ± 0.56%; *p* < 0.05 comparing Im-EO and its baseline) (Figure 5 and Table 1).

Comparing the two antiseptics, although both showed a significant reduction of bacterial viability after immersion in all layers, in layer 3 the 0.2% CHX solution showed more reduced bacterial viability than did the EO (layer 3, Im-0.2% CHX vs. Im-EO, 11.38 ± 5.81% vs. 39.02 ± 17.50%; *p* < 0.001). In contrast, in comparison with the mouthwash application, the Mw-EO obtained lower bacterial viability than the Mw-0.2% CHX, in all layers (*p* < 0.05) (Table 2).

## Discussion

To the best of the author's knowledge, there are no published studies which compare the immediate antibacterial effect of an oral antiseptic applied using the two methods referred to in much of the literature (*ex vivo* passive immersion and *in vivo* active mouthwash) within the same experiment (using the same volunteer and PL-biofilm within one growth period).

In the present series, glass disks were used instead of enamel ones for several reasons. The first, and more important, is that previous research (Netuschil et al., 1998) and own's (unpublished data) revealed that there were no significant differences in the bacterial viability and thickness of the 2-day PL-biofilm formed on enamel or polished glass analyzed with CLSM. This is a very important fact because it is much easier to obtain a regular glass disk in size and thickness than from enamel, being this crucial to the construction of the intraoral splints and the stability of the disks into it. Another reason is the difficulty of analysing the biofilm formed on an enamel disk due to the irregularity (it is not a plane surface) giving distorted images at the CLSM. In addition, although the sterility of the enamel disks could be achieved easily, there always could exist the latent risk of prion diseases, which would bring an important moral dilemma.

### PL-biofilm thickness

The present series showed a constant thickness of the PL-biofilm after all applied situations. The application of the antiseptic, either by immersion or mouthwash, did not change the basal thickness. The previous literature support this situation, no matter the antiseptic solution or the methodology of application used in the studies (Zaura-Arite et al., 2001; Dong et al., 2010; von Ohle et al., 2010; García-Caballero et al., 2013; Quintas et al., 2015), so that a single application of 0.2% CHX or EO does not affect the thickness of a mature biofilm.

### PL-biofilm bacterial viability

In the literature, the mean bacterial viability in a 2-day PL-biofilm oscillated between 60 and 77% (von Ohle et al., 2010; Gu et al., 2012; García-Caballero et al., 2013; Prada-López et al., 2015a; Quintas et al., 2015), the present series showed results in this range (72 and 76% in both baseline situations, before CHX and EO applications, respectively).

The mean bacterial viability after an Im-0.2% CHX ranged from 0.7 to 35.16% in the previous scientific literature (Zaura-Arite et al., 2001; Gosau et al., 2010; von Ohle et al., 2010). Such wide variations might be caused by the different methodologies used in the studies. These differences mainly arise because of a range of factors, such as the antiseptic concentration or the time lapse of application. In their study on PL-biofilm, von Ohle et al. (2010) chose a 0.1% CHX concentration and their immersion times varied between 1 and 10 min. This protocol contrasts with that followed by Zaura-Arite et al. (2001) and Gosau et al. (2010), who evaluated a commercial 0.2% CHX concentration, and selected an immersion time lapse of 1 min. Despite the obvious methodological differences with other series (time of exposure and concentration), the results of the present study in terms of bacterial viability of the PL-biofilm after an Im-0.2% CHX (approximately a 15%) are in accordance with the results reported in the previous literature (Zaura-Arite et al., 2001; Gosau et al., 2010; von Ohle et al., 2010). Respect to the applied CHX protocol, the manufacturer recommendations in terms of time of application were followed (30 s).

Regarding the Mw-0.2% CHX, there are few evaluations of the bacterial viability of the PL-biofilm after an active mouthwash with this antiseptic (García-Caballero et al., 2013; Quintas et al., 2015). In the present series, the bacterial viability was near to 5% which is consistent with that reported in previous studies (García-Caballero et al., 2013; Quintas et al., 2015).

Concerning the mean bacterial viability after an Im-EO, the results found in the literature ranged from 23 to 31% (Dong et al., 2010; Gosau et al., 2010). In these cases, the antiseptic concentration did not vary from one study to another. This is probably one of the reasons why the range is narrower for the EO than for the 0.2% CHX. Gosau et al. (2010) and Dong et al. (2010) immersed a 12 and 48 h-PL-biofilm for 1 min, respectively. The present series showed a mean bacterial viability within the named range (approximately a 27%).

In regard to the assessment of the efficacy of an active Mw-EO, to the best of the author's knowledge, there is only one study in which the bacterial viability of the PL-biofilm has been evaluated (Quintas et al., 2015). This study showed a very low bacterial viability 30 s after the Mw-EO, near to 1%, which is similar to the present results.

Despite the visible lack of previous literature, the results of the present series, in terms of bacterial viability, have a clear interpretation, according to the authors: doing an active mouthwash greatly reduces the bacterial viability of the PL-biofilm, more so than doing an immersion with the same antiseptic. When differentiating between 0.2% CHX and EO, we found doing an Im-0.2% CHX was more effective than doing an Im-EO.

On the other hand, when a mouthwash was done, the EO solution was more effective than the 0.2% CHX. In previous research of the authors (Quintas et al., 2015), the immediate effect of the EO vs. 0.2% CHX has been already presented and discussed in detail.

### PL-biofilm bacterial viability by layers

The distribution of the bacterial viability into the PL-biofilm in the baseline disks was significantly lower in the deepest layers. This distribution pattern of viability, in which vital bacteria overlay non-vital bacteria, has been previously described in other *in vivo* biofilm studies that analyzed bacterial viability in layers (Arweiler et al., 2004; García-Caballero et al., 2013; Prada-López et al., 2015a; Quintas et al., 2015).

After the Im-0.2% CHX, the bacterial viability decreased significantly in all layers. However, there were no differences among the layers (18% outer, 16% middle and 11% inner layer). Zaura-Arite et al. (2001) analyzed the bacterial viability in the different layers after a 1 min immersion in 0.2% CHX showing its efficacy as well, but with wide ranges of values (outer layer = 16–42%, middle layer = 19–55% and inner layer = 21–58%). These data were probably obtained due to the small sample size and the characteristics of the volunteers (three heavy plaque-formers and three light plaque-formers).

On the other hand, the Mw-0.2% CHX reduced the bacterial viability similarly in all layers, being these findings in accordance with those previously described *in situ* studies (García-Caballero et al., 2013; Quintas et al., 2015). This reduction in bacterial viability obtained by mouthwash, as recorded by layers, was higher than the obtained from the immersion method.

In the present series, when applying the EO antiseptic, the bacterial viability of the PL-biofilm was reduced in all layers after the immersion. The outer layer showed significant less viability than the other two (16% in the outer layer vs. 26% in the middle and 39% in the deepest layer). In the same manner, Dong et al. (2010), after a 1-min Im-EO found less bacterial viability in the outer layer (outer layer = 22% vs. middle layer = 34%, and inner layer = 37%). In both studies, a reduction in the bacterial viability was shown, but a different spatial distribution in the bacterial viability compared to their baselines could be seen (Figure 5). While in the baseline sample the bacterial viability decreased from the outer to the inner layers, after an Im-EO, this distribution was inverted, showing an increase in the bacterial viability from the outer to the inner layers. This fact could be explained by the low capacity of the EO solution to penetrate mature biofilms, resulting in a loss of efficacy in the deepest layers of the PL-biofilm. In the contrast, this effect seen after the Im-EO was completely lost after the Mw-EO. The EO applied following the manufacturer's instructions (a single mouthwash with 20 mL for 30 s) was clearly more effective in all layers than the simple immersion, achieving bacterial viability results near to 0% in the three layers.

The results of the present series confirmed that, to properly assess the immediate antibacterial effect of 0.2% CHX and EO, an *in vivo* active mouthwash following the manufacturer's recommendations should be done. This series has also shown that an active mouthwash helped to maximize the efficacy of the 0.2% CHX solution and, mainly, the EO solution compared to a single immersion. In addition, the findings of this investigation suggest cautious interpretation of the results of studies that followed an *ex vivo* antiseptic application (immersion) in PL-biofilm. This previous literature about antimicrobial activity of both 0.2% CHX and EO relies on *in vitro* and *in situ* studies that do not follow proper methodologies, pretending to equate a simple immersion (*ex vivo*) with an active mouthwash (*in vivo*). To some extent, this equation could be considered valid when isolated bacteria are studied. However, when talking about bacteria associated in a more complex structure such as a naturally-formed biofilm (that is 10–1000 times more resistant than bacteria in planktonic phase) (Fine et al., 2001), something more than the simple contact with the antiseptic is needed. This higher activity of the antiseptic when applied as a mouthwash could be due to the hydrodynamic forces that appear in the mouth thank to the action of the tongue, cheeks and other muscles of the oral cavity that contribute to the movement of the mouthwash throughout all the surfaces of the mouth. This movement could achieve something that the passive immersion could not, which is breaking of the surface force of the PL-biofilm, being this crucial for the antiseptic penetration. This is the same theory followed in endodontics with the “hydrodynamic activation” of the antiseptic in the interior of the radicular canal by agitation (Weller et al., 1980; Peeters et al., 2014). This movement achieves to break the surface force of small root canals contributing to maximize the chemical action of the antiseptic (Peeters et al., 2014).

Finally, the authors would like to point out another possible differentiating variable which may condition the antiseptic effectiveness: the temperature. In the *ex vivo* experiment, the antiseptic was at room temperature (between 18 and 20°C). However, when the application was *in vivo*, the temperature of the antiseptic solution rose by several degrees. In future investigations, it would be interesting to study the role that the temperature might play in the antimicrobial effect of the antiseptic.

## Conclusion

The method of application conditioned the antibacterial activity of the 0.2% Chlorhexidine and the Essential Oils on the plaque-like biofilm. The *in vivo* active mouthwash protocol was more effective than the *ex vivo* passive immersion in both antiseptic solutions, conditioning the obtained results. There was more penetration of the antiseptic inside the biofilm with an active mouthwash, especially when the Essential Oils were used.

According to the results of the present study, future investigations on oral antiseptics should take into account the methodology of the application. To obtain a situation as close as possible to the clinical reality, the plaque-like biofilm should be formed *in vivo*. In addition, the antiseptic application should be *in situ*, with an active mouthwash or, at least, take into consideration the role that the movement of the solution may have in the antiseptic antimicrobial activity.

## Author contributions

Conception and design the experiments: IT, JS, DS. Performed the experiments: IP, VQ. Analyzed data: IT, MACDC. Interpretation of the data: IP, VQ. Drafting and revising the manuscript: IP, VQ, MACDC, IT. Final approval: IT, JS, DS. Agreement: IP, VQ, MACDC, JS, DS, IT.

### Conflict of interest statement

The authors declare that the research was conducted in the absence of any commercial or financial relationships that could be construed as a potential conflict of interest.

## Abbreviations

0.2% CHX: solution of 0.2% of Chlorhexidine
EO: Essential Oils
PL-biofilm: plaque-like biofilm
IDODS: intraoral device of overlaid disk-holding splints
Im-0.2%CHX: immersion in 0.2% Chlorhexidine
Im-EO: immersion in Essential Oils
Mw-0.2%CHX: mouthwash with 0.2% Chlorhexidine
Mw-EO: mouthwash with Essential Oils.

## References

[B1] Arweiler N. B. Hellwig E. Sculean A. Hein N. Auschill T. M. (2004). Individual vitality pattern of *in situ* dental biofilms at different locations in the oral cavity. Caries Res. 38, 442–447. 10.1159/000079625 15316188

[B2] Auschill T. M. Arweiler N. B. Netuschil L. Brecx M. Reich E. Sculean A. (2001). Spatial distribution of vital and dead microorganisms in dental biofilms. Arch. Oral. Biol. 46, 471–476. 10.1016/S0003-9969(00)00136-9 11286812

[B3] Auschill T. M. Hein N. Hellwig E. Follo M. Sculean A. Arweiler N. B. (2005). Effect of two antimicrobial agents on early *in situ* biofilm formation. J. Clin. Periodontol. 32, 147–152. 10.1111/j.1600-051X.2005.00650.x 15691343

[B4] Auschill T. M. Hellwig E. Sculean A. Hein N. Arweiler N. B. (2004). Impact of the intraoral location on the rate of biofilm growth. Clin. Oral Investig. 8, 97–101. 10.1007/s00784-004-0255-6 14986070

[B5] Axelsson P. (2004). Preventive Materials, Methods and Programs, 1st Edn. Surrey: Quintessence.

[B6] Corbin A. Pitts B. Parker A. Stewart P. S. (2011). Antimicrobial penetration and efficacy in an *in vitro* oral biofilm model. Antimicrob. Agents Chemother. 55, 3338–3344. 10.1128/AAC.00206-11 21537022 PMC3122404

[B7] Davies D. (2003). Understanding biofilm resistance to antibacterial agents. Nat. Rev. Drug Discov. 2, 114–122. 10.1038/nrd1008 12563302

[B8] Dong W. L. Zhou Y. H. Li C. Z. Liu H. Shang S. H. Pan B. Q. (2010). Establishment and application of an intact natural model of human dental plaque biofilm. Shanghai Kou Qiang Yi Xue 19, 196–201. 20485987

[B9] Fine D. H. Furgang D. Barnett M. L. (2001). Comparative antimicrobial activities of antiseptic mouthrinses against isogenic planktonic and biofilm forms of Actinobacillus actinomycetemcomitans. J. Clin. Periodontol. 28, 697–700. 10.1034/j.1600-051x.2001.028007697.x 11422593

[B10] Fine D. H. Furgang D. Sinatra K. Charles C. McGuire A. Kumar L. D. (2005). *In vivo* antimicrobial effectiveness of an essential oil-containing mouth rinse 12 h after a single use and 14 days' use. J. Clin. Periodontol. 32, 335–340. 10.1111/j.1600-051x.2005.00674.x 15811048

[B11] Fuchslocher Hellemann C. Grade S. Heuer W. Dittmer M. P. Stiesch M. Schwestka-Polly R.. (2013). Three-dimensional analysis of initial biofilm formation on polytetrafluoroethylene in the oral cavity. J. Orofac. Orthop. 74, 458–467. 10.1007/s00056-013-0174-8 24158582

[B12] García-Caballero L. Quintas V. Prada-López I. Seoane J. Donos N. Tomás I. (2013). Chlorhexidine substantivity on salivary flora and plaque-like biofilm: an *in situ* model. PLoS ONE 8:e83522. 10.1371/journal.pone.0083522 24386220 PMC3873939

[B13] Gosau M. Hahnel S. Schwarz F. Gerlach T. Reichert T. E. Burgers R. (2010). Effect of six different peri-implantitis disinfection methods on *in vivo* human oral biofilm. Clin. Oral Implants Res. 21, 866–872. 10.1111/j.1600-0501.2009.01908.x 20666798

[B14] Gu H. Fan D. Gao J. Zou W. Peng Z. Zhao Z.. (2012). Effect of ZnCl_2_ on plaque growth and biofilm vitality. Arch. Oral Biol. 57, 369–375. 10.1016/j.archoralbio.2011.10.001 22071420

[B15] Gunsolley J. C. (2010). Clinical efficacy of antimicrobial mouthrinses. J. Dent. 38(Suppl. 1), S6–S10. 10.1016/S0300-5712(10)70004-X 20621242

[B16] Hannig C. Kirsch J. Al-Ahmad A. Kensche A. Hannig M. Kummerer K. (2013). Do edible oils reduce bacterial colonization of enamel *in situ*? Clin. Oral Investig. 17, 649–658. 10.1007/s00784-012-0734-0 22552590

[B17] Jentsch H. Hombach A. Beetke E. Jonas L. (2002). Quantitative transmission electron microscopic study of dental plaque–an *in vivo* study with different mouthrinses. Ultrastruct. Pathol. 26, 309–313. 10.1080/01913120290104584 12396241

[B18] Netuschil L. Reich E. Unteregger G. Sculean A. Brecx M. (1998). A pilot study of confocal laser scanning microscopy for the assessment of undisturbed dental plaque vitality and topography. Arch. Oral Biol. 43, 277–285. 10.1016/S0003-9969(97)00121-0 9839703

[B19] Pan P. Barnett M. L. Coelho J. Brogdon C. Finnegan M. B. (2000). Determination of the *in situ* bactericidal activity of an essential oil mouthrinse using a vital stain method. J. Clin. Periodontol. 27, 256–261. 10.1034/j.1600-051x.2000.027004256.x 10783839

[B20] Peeters H. H. Iskandar B. Suardita K. Suharto D. (2014). Visualization of the removal of trapped air from the apical region of the straight root canal models generating 2-phase intermittent counter flow during ultrasonically activated irrigation. J. Endod. 40, 857–861. 10.1016/j.joen.2013.10.011 24862717

[B21] Prada-López I. Quintas V. Donos N. Suarez-Quintanilla D. Tomás I. (2015a). Characteristics of *in situ* oral biofilm after 2 and 4 days of evolution. Quintessence Int. 46, 287–298. 10.3290/j.qi.a33402 25642460

[B22] Prada-López I. Quintas V. Tomás I. (2015b). The intraoral device of overlaid disk-holding splintsb as a new *in situ* oral biofilm model. J. Clin. Exp. Dent. 7, 126–132. 10.4317/jced.52093 25810823 PMC4367999

[B23] Quintas V. Prada-López I. Prados-Frutos J. C. Tomás I. (2015). *In situ* antimicrobial activity on oral biofilm: essential oils vs. 0.2 % chlorhexidine. Clin. Oral Investig. 19, 97–107. 10.1007/s00784-014-1224-3 24687247

[B24] Tawakoli P. N. Al-Ahmad A. Hoth-Hannig W. Hannig M. Hannig C. (2013). Comparison of different live/dead stainings for detection and quantification of adherent microorganisms in the initial oral biofilm. Clin. Oral Investig. 17, 841–850. 10.1007/s00784-012-0792-3 22821430

[B25] Varoni E. Tarce M. Lodi G. Carrassi A. (2012). Chlorhexidine (CHX) in dentistry: state of the art. Minerva Stomatol. 61, 399–419. 22976567

[B26] Vitkov L. Hermann A. Krautgartner W. D. Herrmann M. Fuchs K. Klappacher M.. (2005). Chlorhexidine-induced ultrastructural alterations in oral biofilm. Microsc. Res. Techniq. 68, 85–89. 10.1002/jemt.20238 16228984

[B27] von Ohle C. Gieseke A. Nistico L. Decker E. M. DeBeer D. Stoodley P. (2010). Real-time microsensor measurement of local metabolic activities in *ex vivo* dental biofilms exposed to sucrose and treated with chlorhexidine. Appl. Environ. Microbiol. 76, 2326–2334. 10.1128/AEM.02090-09 20118374 PMC2849229

[B28] Weller R. N. Brady J. M. Bernier W. E. (1980). Efficacy of ultrasonic cleaning. J. Endod. 6, 740–743. 10.1016/S0099-2399(80)80185-3 6935384

[B29] WHO (1997). Oral health surveys, basic methods, in World Health Organization, 4th Edn., ed Van Palenstein Helderman W. H. (Geneva: WHO), 36–38.

[B30] Zaura-Arite E. van Marle J. ten Cate J. M. (2001). Conofocal microscopy study of undisturbed and chlorhexidine-treated dental biofilm. J. Dent. Res. 80, 1436–1440. 10.1177/00220345010800051001 11437215

